# Prognostic role of left atrial strain and its combination index with transmitral E-wave velocity in patients with atrial fibrillation

**DOI:** 10.1038/srep17318

**Published:** 2016-02-01

**Authors:** Po-Chao Hsu, Wen-Hsien Lee, Chun-Yuan Chu, Hung-Hao Lee, Chee-Siong Lee, Hsueh-Wei Yen, Tsung-Hsien Lin, Wen-Chol Voon, Wen-Ter Lai, Sheng-Hsiung Sheu, Ho-Ming Su

**Affiliations:** 1 Division of Cardiology, Department of Internal Medicine, Kaohsiung Medical University Hospital, Kaohsiung Medical University, Kaohsiung, Taiwan; 2 Department of Internal Medicine, Kaohsiung Municipal Hsiao-Kang Hospital, Kaohsiung Medical University, Kaohsiung, Taiwan; 3 Faculty of Medicine, College of Medicine, Kaohsiung Medical University, Kaohsiung, Taiwan

## Abstract

Left atrial (LA) strain can reflect LA remodeling and is reduced in atrial fibrillation (AF) patients with prior stroke. This study sought to examine the ability of LA strain in predicting subsequent stroke event in AF and also evaluated whether E/LA strain could predict cardiovascular (CV) events in these patients. In 190 persistent AF patients, we performed comprehensive echocardiography with assessment of LA strain. There were 69 CV events including 19 CV death, 32 hospitalizations for heart failure, 3 myocardial infarctions, and 15 strokes during an average follow-up of 29 months. Multivariate analysis showed old age, chronic heart failure, increased left ventricular (LV) mass index, and increased E/LA strain were associated with CV events and decreased LA strain was associated with subsequent stroke event. The addition of E/LA strain and LA strain to a model containing CHA_2_DS_2_-VASc score and LV function significantly improved the values in predicting CV events and subsequent stroke event, respectively. In conclusion, E/LA strain and LA strain were respectively useful in predicting CV events and subsequent stroke event in AF. E/LA strain and LA strain could provide incremental values for CV outcome and subsequent stroke outcome prediction over conventional clinical and echocardiographic parameters in AF, respectively.

Atrial fibrillation (AF) is the most common form of cardiac arrhythmia and patients with AF are associated with an increased risk of stroke, heart failure, and cardiovascular (CV) mortality^1^,^2^. The traditionally echocardiographic parameters associated with an increased risk of stroke are increased left atrial (LA) dimension, decreased LA appendage flow velocity, and proof of thrombi or spontaneous echo contrast^3^,^4^.

Two-dimensional echocardiographic speckle tracking can accurately measure left ventricular strain and strain rate^5^,^6^. This imaging method has also been used for the assessment of LA function^7^,^8^. LA remodeling, including structural, functional, and electrical changes is frequently noted in patients with AF. Severely impaired LA strain may reflect more advanced LA remodeling^9^,^10^. LA strain is also correlated with LA fibrosis in patients irrespective of the presence of AF or not^11^,^12^. Additionally, LA strain is significantly reduced in AF patients with prior stroke compared to those without prior stroke^13^. LA strain and LA volume may provide complementary information on structural changes of the left atrium, but it is speculated that LA strain may be a more sensitive parameter of changes in LA wall structure. However, there is no study to evaluate whether LA strain is a useful predictor of subsequent stroke in patients with AF. In addition, several echocardiographic combination indices generated by the ratio of transmitral E-wave velocity (E) to left ventricular diastolic parameters can effectively predict CV outcomes^14^,^15^,^16^,^17^. LA strain has been demonstrated to be associated with left ventricular diastolic function^18^. We hypothesize the combination index, E/LA strain, is also a useful parameter in predicting CV events in AF patients. Hence, this study was designed to investigate whether E/LA strain and LA strain could respectively predict adverse CV events and subsequent stroke event in patients with AF.

## Methods

### Study patients

This observational cohort study prospectively and consecutively included patients with persistent AF referred for echocardiographic examinations at Kaohsiung Municipal Hsiao-Kang Hospital from April 2010 to June 2012. Persistent AF was defined as AF lasting for at least 7 days. Patients with moderate and severe mitral stenosis (n = 5), moderate and severe aortic stenosis or regurgitation (n = 4), severe mitral regurgitation (n = 5), and inadequate echocardiographic visualization (n = 11) were excluded. Additionally, four patients who had no beat fulfilling the requirements of index beat in the stored cardiac cycles were also excluded. Finally, 190 AF patients were included in this study. The study protocol was approved by our Institutional Review Board and all enrolled patients gave written, informed consent.

### Echocardiographic evaluation

The echocardiographic examination was performed by one experienced cardiologist with a VIVID 7 (General Electric Medical Systems, Horten, Norway). The cardiologist was blinded to the clinical data. Pulsed tissue Doppler imaging was obtained with the sample volume placed at the lateral and septal corners of the mitral annulus from the apical 4-chamber view. Early diastolic mitral annulus velocity (E’) was averaged from septal and lateral ones. The wall filter settings were adjusted to exclude high-frequency signals and the gain was minimized. Left ventricular ejection fraction (LVEF) was measured using the modified Simpson’s method. Left atrial volume was measured using the biplane area-length method^19^. Left atrial volume index (LAVI) was calculated by dividing left atrial volume by body surface area.

### LA strain measurement

The endocardial border was manually defined using a point-and-click technique. An epicardial surface tracing was automatically generated by the system, creating a region of interest, which was manually adjusted to cover the full thickness of left atrium. Time-strain plot was produced automatically by the software. Global LA strain during the reservoir phase was estimated by taking the average of longitudinal strain data obtained from the apical four-chamber and two chamber projections^10^,^13^,^20^. Data from a total of 12 LA segments (annular, mid, and superior segments along the septal, lateral, anterior, and inferior LA walls using apical four-chamber and two-chamber images) were averaged to determine global longitudinal LA strain at the end of left ventricular ejection (LA reservoir phase). Assessment of LA strain was accepted if at least four of the six LA segments in each view could be measured clearly. We used cine loops to determine which beat would be calculated. The raw ultrasonic data, including 15 consecutive beats from the apical four-chamber and two-chamber views, was recorded and analyzed offline using EchoPAC software (EchoPAC version 08; GE-Vingmed Ultrasound AS GE Medical Systems).

Left ventricular dimensions, LVEF, LAVI, and LA strain were measured from the index beat method^21^,^22^. Because their measurements were easy and rapid, the E, E-wave deceleration time, and E’ were obtained from five beats^23^ and then averaged for later analysis. If the cardiac cycle length was too short to complete the diastolic process, this beat was skipped. Thus, the selection of E, E-wave deceleration time, and E’ was not always consecutive. In addition, heart rate was determined from five consecutive beats.

### Collection of demographic, medical, and laboratory data

Demographic and medical data including age, gender and history of diabetes mellitus, hypertension, coronary artery disease, stroke, and chronic heart failure were obtained from medical records or interviews with patients. Laboratory data including total cholesterol and triglyceride were also collected. In addition, information regarding patient medications during the study period was obtained from medical records.

### Definition of CV events

CV events were defined as CV mortality, hospitalization for heart failure, myocardial infarction, and stroke. Hospitalization for heart failure was defined as admission due to dyspnea with chest radiographic evidence of pulmonary congestion and treatment with intravenous diuretics. CV events were ascertained and adjudicated by two cardiologists with disagreement resolved by adjudication from a third cardiologist from the hospital course and medical record. If patients had multiple CV events, only the first event was coded. However, if patients died after episodes of heart failure, myocardial infarction, or stroke during the same admission, they were coded as CV death. In patients reaching the study end points, they were followed until the first episode of adverse events. The other patients were followed until March 2014.

### Statistical analysis

SPSS 18.0 software (SPSS, Chicago, IL, USA) was used for statistical analysis. Data were expressed as mean ± standard deviation, percentage, or median (25^th^–75^th^ percentile) for follow-up period. Continuous and categorical variables among groups were compared by one-way analysis of variance (ANOVA) followed by a post hoc test adjusted with a Bonferroni correction and Chi-square test, respectively. The relationship between two continuous variables was assessed by a bivariate correlation method (Pearson’s correlation). The significant variables in the univariate analysis were selected for multivariate analysis. Time to the adverse events and covariates of risk factors were modeled using a Cox proportional hazards model with forward selection. A significant improvement in model prediction was based on the −2 log likelihood ratio statistic, which followed a difference in −2 log likelihood value and the P value was based on the incremental value compared with the previous model. Kaplan-Meier survival plots were calculated from baseline to time of adverse events and compared using the log-rank test. Stepwise multiple linear regression analysis was employed to identify the determinants of LA strain and E/LA strain. All tests were 2-sided and the level of significance was established as P < 0.05.

## Results

Table 1 shows the comparison of clinical and echocardiographic characteristics according to the tertile of LA strain. There were significant differences in the age, prevalence of coronary artery disease and chronic heart failure, CHA_2_DS_2_-VASc score, heart rate, anticoagulant use, LAVI, left ventricular end-systolic and end-diastolic dimensions, LVEF, E’, E/E’, and LA strain among patients with different tertiles.

The follow-up period to CV events was 29 (25^th^–75^th^ percentile: 18–36) months in all patients. Sixty-nine CV events were documented during the follow-up period, including CV death (n = 19), hospitalization for heart failure (n = 32), myocardial infarction (n = 3), and stroke (n = 15). A Cox proportional hazards regression analysis for CV events is shown in Table 2. In the multivariate analysis, old age, the presence of chronic heart failure, increased LVMI, and increased E/LA strain (hazard ratio [HR], 1.182; 95% confidence interval [CI], 1.086 to 1.286; P < 0.001) were independently associated with increased CV events.

The follow-up period to subsequent stroke event was 30 (25^th^–75^th^ percentile: 23–41) months in all patients. Eighteen subsequent stroke events were documented during the follow-up period. A Cox proportional hazards regression analysis for subsequent stroke event is shown in Table 3. In the multivariate analysis, only decreased LA strain (HR, 0.844; 95% CI, 0.745 to 0.955; P = 0.007) was independently associated with increased subsequent stroke event.

**Figure 1:**
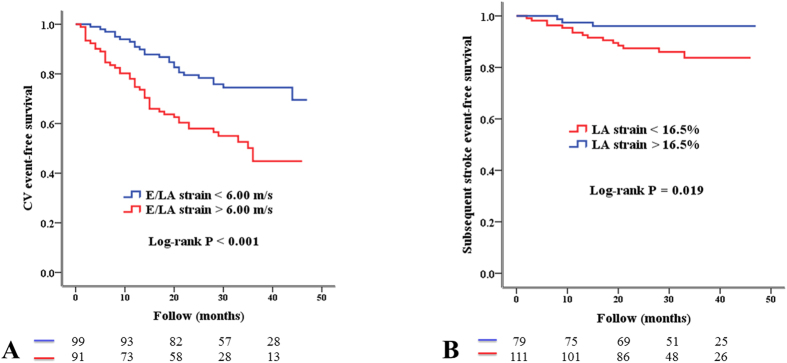
Kaplan-Meier analysis of cardiovascular (CV) event-free survival according to the ratio of transmitral E-velocity (E) to left atrial (LA) strain <6.00 or >6.00 m/s (A) and of subsequent stroke event-free survival according to LA strain >16.50 or <16.50% (B).

To find the appropriate cut-off values to E/LA strain and LA strain as predictors of the outcomes, we created several models using different cut-off values of E/LA strain and LA strain. Using the −2 log likelihood value to select the model with the best performance, the model using E/LA strain >6.00 m and LA strain <16.50% had the best performance in predicting the adverse events. Figure 1 illustrates the Kaplan-Meier curves for CV event-free survival and subsequent stroke-free survival in study patients.

The incremental values of E/LA strain and LA strain in outcome prediction are shown in Table 4. The addition of E/LA strain and LA strain to a Cox model containing CHA_2_DS_2_-VASc score, LAVI, LVEF, and E/E’ significantly improved the values in predicting CV events and subsequent stroke event respectively. However, the addition of LA strain and E/LA strain to a Cox model containing CHA_2_DS_2_-VASc score, LAVI, LVEF, and E/E’ did not improve the values in predicting CV events and subsequent stroke event, respectively.

## Discussion

In the present study, we evaluated the association of E/LA strain with CV events and LA strain with subsequent stroke event in patients with AF. We found that increased E/LA strain and decreased LA strain were independently associated with an increase in CV events and subsequent stroke event in AF, respectively. The E/LA strain and LA strain could respectively add significant incremental values beyond the conventional clinical and echocardiographic parameters in prediction of CV events and subsequent stroke event.

Diastolic dysfunction increases left ventricular filling pressure and is the primary mechanism responsible for the clinical findings of heart failure. Echocardiography is the most valuable tool for the noninvasive evaluation of diastolic function^24^. Several approaches on the basis of Doppler modalities have been proposed as useful methods for the evaluation of left ventricular diastolic function^25^,^26^. By combining these parameters with those obtained from the mitral flow curve, a more precise estimation of left ventricular filling pressure has been achieved^27^,^28^. E/E’ was reported to be related well to mean pulmonary capillary wedge pressure^28^ and the ratio of E to global diastolic strain rate had also been proposed as a marker of elevated left ventricular filling pressure^29^. Ersbøll M *et al.* showed the ratio of E to global early diastolic strain rate (E’sr) was independently associated with an adverse outcome in patients with myocardial infarction^14^. Our previous study also showed that E/E’sr was a useful parameter in predicting adverse cardiac events in AF^17^. LA strain has demonstrated to be associated with left ventricular diastolic function^18^. Hence, E/LA strain should be a good parameter in the evaluation of left ventricular filling pressure. In fact, the present study showed increased E/LA strain was highly correlated with increased E/E’, a good parameter of left ventricular filling pressure^28^, in the multivariate analysis. The LA strain has been reported to be useful in predicting CV prognosis in AF patients with acute embolism^30^. In the present study, although decreased LA strain and increased E/LA strain were associated with CV events in the univariate analysis, only E/LA strain was still associated with adverse CV events after multivariate analysis. Furthermore, the addition of E/LA strain, but not LA strain, to a Cox model consisting of conventional clinical and echocardiographic parameters could cause an improvement in prediction of adverse CV events. Hence, the combination index, E/LA strain, might be more useful in predicting poor CV prognosis in AF patients than LA strain.

Several traditionally echocardiographic parameters including increased LA dimension, decreased LA appendage flow velocity, and proof of thrombi or spontaneous echo contrast were associated with an increased risk of stroke^3^,^4^. Recently, LA strain was reported to decrease proportionately with increasing CHADS_2_ score and was an independent predictor of prior stroke^13^,^31^. Shih *et al.* found decreased LA strain and strain rate were independently associated with prior stroke, but E and E/E’ were not^13^. The finding might indicate that stroke event was correlated better with LA-related parameters. However, this study was a cross-sectional study without long-term outcome data. In addition, it has also been proven that in patients with CHADS_2_ scores ≦1, LA strain is an independent predictor of prior stroke, when adjusted for LA size, LVEF, and left ventricular mass^32^. In a recent observational study, LA strain provided incremental value for embolism risk stratification over CHA_2_DS_2_-VASc score in patients with AF^30^. In the present study, we similarly found decreased LA strain was correlated with increased CHA_2_DS_2_-VASc score in the univariate analysis and further found decreased LA strain was significantly associated with subsequent stroke event even after adjustment for many important echocardiographic parameters. In addition, the addition of LA strain to a Cox model consisting of conventional clinical and echocardiographic parameters could cause an improvement in prediction of subsequent stroke event. Hence, impaired LA strain may be useful in predicting subsequent stroke event in AF patients.

The underlying mechanism of the association between impaired LA strain and subsequent stroke event remains unclear. Previous studies have found there is an association among reduced LA strain, LA fibrosis, lower LA appendage flow velocity, and LA appendage thrombus^11^,^33^. We hypothesize that LA fibrosis diminishes LA compliance during the LA reservoir phase, which causes blood flow stasis in the left atrium. Therefore, reduced LA strain may contribute to an increased risk for subsequent stroke. However, E/LA strain was not associated with subsequent stroke event, which might also indicate that LA strain itself predicted future stroke event better than parameters of left ventricular filling pressure. On the contrary, E/LA strain might predict future CV events better than LA strain itself because of the close association between E/LA strain and left ventricular filling pressure.

### Study limitations

There were several limitations to this study. First, since the subjects of this study were already being evaluated for heart disease by echocardiography, it was susceptible to selection bias, making findings potentially less generalized, and making study sample size relatively small. Second, two-dimensional speckle tracking echocardiography could generate LA strain and strain rate curves among different LA cycle phases. In this study, only longitudinal LA strain during the reservoir phase was measured and analyzed. Third, percentage of anticoagulant use was relatively low in our study. According to the literature published by Taiwan Stroke Registry^34^, only 28.3% were prescribed traditional anticoagulant agent among patients with AF. However, this condition may have gradually improved after new anticoagulant agent use in Taiwan. Finally, echocardiographic parameters in our study were analyzed only by a single operator without core lab readings and outcomes were obtained from medical records only.

## Conclusions

In patients with AF, E/LA strain and LA strain were useful in predicting adverse CV events and subsequent stroke event, respectively. Additionally, E/LA strain and LA strain could respectively provide incremental values for CV outcome and subsequent stroke outcome prediction over conventional clinical and echocardiographic parameters in AF.

## Additional Information

**How to cite this article**: Hsu, P.-C. *et al.* Prognostic role of left atrial strain and its combination index with transmitral E-wave velocity in patients with atrial fibrillation. *Sci. Rep.* **6**, 17318; doi: 10.1038/srep17318 (2016).

## Figures and Tables

**Table 1:** Comparison of clinical and echocardiographic characteristics according to tertile of LA strain.

Characteristics	Tertile 1 (LA strain>17.75%, n = 63)	Tertile 2 (LA strain:13.60–17.75%, n = 63)	Tertile 3 (LA strain<13.60%, n = 64)	P	All patients(n = 190)
Age (year)	68 ± 10	71 ± 9	72 ± 10*	0.025	70 ± 10
Male gender	41 (65%)	39 (64%)	48 (75%)	0.438	67%
Diabetes mellitus	18 (29%)	20 (32%)	14(22%)	0.444	27%
Hypertension	40 (63%)	42 (67%)	43 (67%)	0.894	66%
CAD	4 (6%)	3 (5%)	13 (20%)*#	0.007	11%
Stroke	7 (11%)	11 (17%)	15 (23%)	0.186	17%
CHF	8 (13%)	19 (30%)*	27 (42%)*	0.001	29%
CHA_2_DS_2_-VASc score	2.83 ± 1.80	3.56 ± 1.83	3.80 ± 1.66*	0.006	3.39 ± 1.80
SBP (mmHg)	133 ± 22	128 ± 17	136 ± 19	0.085	133 ± 20
DBP (mmHg)	78 ± 12	76 ± 12	78 ± 14	0.569	77 ± 12
Heart rate (min^−1^)	77 ± 17	86 ± 20*	86 ± 22*	0.018	83 ± 20
Body mass index (kg/m^2^)	26.6 ± 3.8	26.3 ± 4.4	25.7 ± 4.2	0.450	26.3 ± 4.1
Triglyceride (mg/dL)	123 ± 95	111 ± 56	133 ± 80	0.421	123 ± 79
Total cholesterol (mg/dL)	169 ± 32	174 ± 34	176 ± 41	0.576	173 ± 36
Medications					
ACEI and/or ARB use	32 (51%)	34 (54%)	38 (59%)	0.617	54%
β-blocker use	29 (46%)	27 (43%)	27 (42%)	0.897	44%
CCB use	24 (38%)	26 (41%)	15 (23%)	0.077	34%
Diuretics use	25 (40%)	22 (35%)	33 (52%)	0.147	42%
Antiplatelet use	41 (65%)	35 (56%)	36 (56%)	0.479	59%
Anticoagulant use	11 (17%)	20 (32%)	24 (38)*	0.038	29%
Echocardiographic data					
LAVI (ml/m^2^)	43 ± 16	44 ± 15	56 ± 24*#	<0.001	48 ± 20
LVEDD (mm)	50 ± 7	51 ± 7	54 ± 9*	0.005	52 ± 8
LVESD (mm)	32 ± 7	36 ± 8	39 ± 11*	<0.001	36 ± 9
LVMI (g/m^2^)	127 ± 36	129 ± 35	155 ± 43*#	0.001	137 ± 40
LVEF (%)	62 ± 10	56 ± 12	46 ± 16*#	<0.001	55 ± 14
E (cm/s)	98 ± 21	96 ± 21	96 ± 25	0.859	97 ± 23
EDT (ms)	152 ± 45	143 ± 38	152 ± 53	0.451	149 ± 46
E’ (cm/s)	10.3 ± 2.5	8.9 ± 1.7*	7.4 ± 1.9*#	<0.001	8.8 ± 2.4
E/E’	10.1 ± 3.8	11.3 ± 3.8	13.8 ± 5.2*#	<0.001	11.8 ± 4.5
LA strain (%)	22.02 ± 3.73	15.65 ± 1.21*	11.14 ± 1.98*#	<0.001	16.24 ± 5.14
E/LA strain (m/s)	4.53 ± 1.14	6.21 ± 1.53*	8.81 ± 2.61*#	<0.001	6.53 ± 2.57

ACEI: angiotensin converting enzyme inhibitor; ARB: angiotensin II receptor blocker; CAD: coronary artery disease; CCB: calcium channel blocker; CHF: chronic heart failure; DBP: diastolic blood pressure; EDT: E wave deceleration time; LA: left atrial; LAVI: left atrial volume index; LVEDD: left ventricular end-diastolic dimension; LVEF: left ventricular ejection fraction; LVESD: left ventricular end-systolic dimension; LVMI: left ventricular mass index; SBP: systolic blood pressure.

^*^P < 0.05 compared with tertile 1; ^#^P < 0.05 compared with tertile 2.

**Table 2:** Predictors of cardiovascular events (cardiovascular mortality, hospitalization for heart failure, myocardial infarction, and stroke) using Cox proportional hazards model.

Parameter	Univariate		Multivariate (Forward)	
	HR (95% CI)	*P*	HR (95% CI)	*P*
Age	1.048 (1.022–1.075)	<0.001	1.044 (1.017–1.071)	0.001
Male gender	1.204 (0.715–2.026)	0.485		
Diabetes mellitus	1.190 (0.706–2.005)	0.513		
Hypertension	0.788 (0.486–1.278)	0.335		
CAD (%)	1.910 (1.002–3.642)	0.049		
Stroke (%)	0.969 (0.508–1.848)	0.923		
CHF (%)	3.025 (1.883–4.858)	<0.001	2.308 (1.400–3.807)	0.001
SBP (mmHg)	1.004 (0.991–1.018)	0.531		
DBP (mmHg)	1.005 (0.983–1.027)	0.661		
Heart rate (min^−1^)	1.004 (0.993–1.016)	0.465		
Body mass index (kg/m^2^)	0.900 (0.841–0.962)	0.002		
Triglyceride (mg/dL)	0.998 (0.994–1.002)	0.393		
Total cholesterol (mg/dL)	0.994 (0.986–1.002)	0.119		
Medications				
ACEI and/or ARB use (%)	1.126 (0.698–1.817)	0.626		
β-blocker use (%)	1.015 (0.631–1.632)	0.950		
CCB use (%)	1.125 (0.685–1.850)	0.642		
Diuretics use (%)	1.902 (1.184–3.058)	0.008		
Antiplatelet use (%)	1.064 (0.656–1.726)	0.803		
Anticoagulant use (%)	0.975 (0.574–1.654)	0.924		
Echocardiographic data				
LAVI (ml/m^2^)	1.012 (0.999–1.024)	0.065		
LVEDD (mm)	1.031 (0.999–1.064)	0.056		
LVESD (mm)	1.039 (1.013–1.067)	0.004		
LVMI (g/m^2^)	1.013 (1.007–1.019)	<0.001	1.011 (1.005–1.016)	<0.001
LVEF (%)	0.968 (0.953–0.984)	<0.001		
E (cm/s)	1.009 (0.999–1.019)	0.070		
EDT (ms)	1.004 (1.000–1.008)	0.037		
E’ (cm/s)	0.783 (0.704–0.872)	<0.001		
E/E’	1.099 (1.060–1.140)	<0.001		
LA strain (%)	0.871 (0.820–0.925)	<0.001		
E/LA strain (m/s)	1.217 (1.131–1.310)	<0.001	1.182 (1.086–1.286)	<0.001

HR: hazard ratio; CI: confidence interval; other abbreviations as in Table 1.

**Table 3:** Predictors of subsequent stroke event using Cox proportional hazards model.

Parameter	Univariate		Multivariate (Forward)	
	HR (95% CI)	*P*	HR (95% CI)	*P*
Age	1.036 (0.986–1.088)	0.162		
Male gender	1.288 (0.459–3.614)	0.631		
Diabetes mellitus	1.283 (0.422–3.898)	0.661		
Hypertension	0.813 (0.315–2.098)	0.669		
CAD (%)	2.038 (0.271–15.320)	0.489		
Stroke (%)	1.920 (0.682–5.405)	0.217		
CHF (%)	1.906 (0.551–6.572)	0.309		
SBP (mmHg)	1.006 (0.981–1.031)	0.638		
DBP (mmHg)	0.983 (0.940–1.027)	0.436		
Heart rate (min^−1^)	0.996 (0.972–1.020)	0.725		
Body mass index (kg/m^2^)	0.908 (0.803–1.028)	0.126		
Triglyceride (mg/dL)	0.998 (0.992–1.005)	0.667		
Total cholesterol (mg/dL)	0.992 (0.977–1.007)	0.280		
Medications				
ACEI and/or ARB use (%)	1.002 (0.3956–2.538)	0.997		
β–blocker use (%)	0.788 (0.305–2.033)	0.622		
CCB use (%)	1.298 (0.462–3.641)	0.621		
Diuretics use (%)	1.115 (0.432–2.877)	0.822		
Antiplatelet use (%)	0.822 (0.324–2.085)	0.680		
Anticoagulant use (%)	1.623 (0.628–4.1934)	0.318		
Echocardiographic data				
LAVI (ml/m^2^)	1.020 (1.000–1.039)	0.047		
LVEDD (mm)	0.993 (0.930–1.059)	0.827		
LVESD (mm)	0.986 (0.931–1.043)	0.619		
LVMI (g/m^2^)	1.011 (1.000–1.022)	0.050		
LVEF (%)	0.984 (0.953–1.015)	0.305		
E (cm/s)	1.000 (0.980–1.021)	0.969		
EDT (ms)	1.001 (0.992–1.011)	0.818		
E’ (cm/s)	0.786 (0.633–0.976)	0.029		
E/E’	1.086 (1.006–1.174)	0.036		
LA strain (%)	0.841 (0.745–0.950)	0.005	0.844 (0.745–0.955)	0.007
E/LA strain (m/s)	1.187 (1.013–1.391)	0.034		

HR: hazard ratio; CI: confidence interval; other abbreviations as in Table 1.

**Table 4:** Incremental values of E/LA strain and LA strain in relation to CV events and subsequent stroke event.

Parameters	CV events		Subsequent stroke event	
	Difference in −2 loglikelihood value	P	Difference in −2 loglikelihood value	P
Model 1: CHA_2_DS_2_-VASc score				
Model 2: model 1 + LAVI, LVEF, E/E’	88.799	<0.001	16.461	<0.001
Model 3: model 2 + E/LA strain	5.383	0.020	0.694	0.405
Model 4: model 2 + LA strain	2.764	0.096	5.589	0.018

P value was based on the difference in −2 log likelihood value compared with the previous model.

CV: cardiovascular; LA: left atrial; LVEF: left ventricular ejection fraction.
